# Public health risks of raw milk consumption: Lessons from a case of paediatric hemolytic uremic syndrome

**DOI:** 10.14745/ccdr.v49i09a03

**Published:** 2023-09-01

**Authors:** Angela Silveira, Julia Pinheiro Carvalho, Lawrence Loh, Michael Benusic

**Affiliations:** 1 University of Saskatchewan, Saskatoon, SK; 2 Saskatchewan Health Authority, Saskatoon, SK; 3 Université de Montréal, Montréal, QC; 4 University of Toronto, Dalla Lana School of Public Health, Toronto, ON; 5 The College of Family Physicians, Mississauga, ON; 6 Island Health, Victoria, BC

**Keywords:** raw milk, raw dairy, unpasteurized, hemolytic uremic syndrome, EHEC, illegal milk consumption

## Abstract

Pasteurization of raw milk is mandatory before sale in Canada and has been demonstrated to reduce the risk of food-borne illness associated with milk consumption. Consumption of raw milk sparks urgent concern from a public health perspective since it has been linked to numerous outbreaks by enteric organisms, particularly *Escherichia coli-*related illnesses and complications in pediatric populations. The sale and distribution of raw milk is illegal in Canada, based on these significant health risks, but growing popular interest and trends in consuming raw dairy products reflect changes in consumer preferences. Although the consumption of raw milk has been an ongoing issue, this new trend is alarming and action is needed to prevent serious consequences as seen in children and other populations with reduced immunity such as the elderly and pregnant people. This commentary explores key issues identified by a local public health unit during the investigation of a recent paediatric case of hemolytic uremic syndrome related to an *E. coli* O157:H7 infection that occurred within the context of consumption of raw milk. The main objective of this article is to highlight that the health risks and sequelae associated with consumption of raw milk far outweigh any potential benefits, with severe consequences particularly among children. Data and health impacts, distribution, regulation, pasteurization and proposed practice recommendations are also identified and discussed.

## Clinical case

An eight-year-old male child presented to a hospital emergency department in southern Ontario with low-grade fever, abdominal pain and bloody diarrhea over four days. He reported no other sick contacts and immunization status was up to date. A detailed food history done in the emergency department identified recent consumption of raw, unpasteurized milk. Baseline testing revealed thrombocytopenia, hemolytic anemia and elevated leukocytes, and subsequent stool cultures were positive for *Escherichia coli* O157:H7.

The patient was admitted and started with intravenous cefuroxime and metronidazole. Despite antibiotic therapy, bloody stools and abdominal pain persisted following admission. On day 6, the patient started to deteriorate rapidly, exhibiting neurological symptoms and renal failure, which prompted intensive care unit admission and a colonoscopy, which identified pseudomembranous exudate.

Hemolytic uremic syndrome (HUS) was subsequently diagnosed given the clinical picture and findings highly suggestive of enterohaemorrhagic *E. coli* (EHEC) ((1)). Enterohaemorrhagic *E. coli* is a subtype of Shiga toxin-releasing *E. coli*. Shiga toxin-releasing *E. coli* strains are a prime concern to food establishments because the disease can progress to HUS, a potentially fatal illness ((1)). The discontinuation of antibiotics allowed the patient to gradually improve with supportive treatment and fluid resuscitation.

A concurrent public health investigation in the same health unit also identified three other cases of Shiga toxin-releasing *E. coli* infections from individuals between five and 25 years of age also associated with the consumption of raw milk. Confirmation arose through molecular testing of stool samples from all four cases and a sample of raw milk kept by one of the cases’ parents. The raw milk in question had been obtained from an illicit network that distributed three-litre glass jars from a delivery vehicle at various points within the local community. Further investigation failed to identify the distributor or the production facility that provided the product.

## Background

*Escherichia coli* is a diverse group of bacteria categorized into six pathotypes referred to as diarrheagenic *E. coli*. Infection with a particular *E. coli* strain is attributed to developing HUS, which presents with anemia, severe renal failure, seizures and risk of death, as was seen in the presented case.

Typically occurring in childhood, HUS is commonly caused by *E. coli* O157:H7 Shiga toxin-producing bacteria ((1)). In cases of *E. coli* O157:H7 gastroenteritis, bloody diarrhea occurs 3–4 days after ingestion of contaminated food, such as raw milk. Patients may also report severe abdominal pain and painful defecation, which can help to distinguish *E. coli* O157:H7 from other causes of bacterial gastroenteritis ((1)). Treatment is typically supportive. Antibiotic therapy is contraindicated since it increases the risk of HUS.

The case presented here is a prime example of how infection with *E. coli* can be traced to the consumption of raw dairy products ((2)). In a recent systematic review, the majority of HUS cases (83%) in North America from 2007 to 2020 can be attributed to 20 outbreaks due to raw dairy consumption, with 14 of these outbreaks involving raw milk consumption. From these 20 outbreaks, 530 illnesses were reported, with 98 paediatric cases (confirmed and suspected) ((2)). As in this case, the risk of acute and chronic renal failure with HUS is high. Rare and serious complications were included in six cases of HUS ((2)), similar to the case presented in this article. To reduce the risk of infection, it is important to improve the measures of control by enhancing the management and preventing the transmission of *E. coli* strains among animals, environment and humans ((3)). Despite widespread public health messaging around the dangers of consuming raw milk, a Canadian Community Health Survey (in-person and telephone interviews) revealed that 3.6% of participants had consumed raw milk in the seven days prior to the interview ((2)). Parents providing their children with raw milk should be educated about the risk of HUS.

### Data and health impact of raw milk consumption

Between 2005 and 2013, 263 confirmed cases of enteric and zoonotic illnesses in Canada were attributed to the consumption of raw milk products ((3)). This number is likely an underestimate, as the vast majority of enteric illnesses often do not present to health care or are tested to a confirmatory extent; literature has identified that there may be nearly 25 times the number of unreported cases of illness as compared to confirmed cases in at least one jurisdiction in the United States ((4)). According to a 2017 study, unpasteurized dairy products cause 840 times more illnesses and 45 times more hospitalizations than pasteurized products, making raw milk a dangerous food ((4)).

Consumption of raw milk is often driven by the perception that consumption can “boost the immune system” and “tastes better than pasteurized milk” and some links that raw milk products can prevent atopy in children and adults ((5)). These perceptions are not evidence-based and a review of the literature, including reports of outbreaks, suggests that the overall risk of consuming these products outweigh the health claims, particularly among those with decreased immunity (children and the elderly) and pregnant people. In North America, from 2000 to 2009, there were a total of 26 outbreaks related to raw milk resulting in an estimated 545 illnesses, more than 23 hospitalizations and 7 infant deaths ((6)). In Ontario alone, from 2005 to 2007, there were 92 cases of illness associated with the consumption of raw milk and raw milk cheese ((3)). These numbers likely represent an underestimate of the true extent of the problem as many of these illnesses are underreported and do not present to the hospital unless symptoms are severe.

While consumption of raw milk is not prohibited, it is illegal to sell, deliver or distribute raw milk in Ontario under the *Milk Act*, a regulatory context mirrored in other Canadian provinces through similar acts (**Table 1**). This means that interested consumers continue to access raw milk products through illegal distribution networks ((6)). The obscure nature of such networks naturally limits the amount of data on the extent and magnitude of distribution ((6)).

**Table 1:** Relevant provincial regulations in respect of mandatory pasteurization^a^

Province	Provincial regulations
British Columbia	*Milk Industry Act*
Alberta	*Dairy Industry Act 2000*
Saskatchewan	*The Milk Compositional Standards Regulation*
Manitoba	*The Dairy Act*
Ontario	*Milk Act*
Québec	*Food Products Act*
New Brunswick	*Dairy Products Regulation*
Nova Scotia	*Dairy Industry Act*
Prince Edward Island	*Dairy Producers Act*
Newfoundland and Labrador	*Milk Regulations*

^a^ Reference 7

### Distribution, regulation and pasteurization

A 2018 study found raw milk to be responsible for almost three times more hospitalizations than any other food-borne illness ((8)). With health implications in mind, it is essential for consumers and policy-makers alike to understand the need for pasteurization. Laws requiring pasteurization can be traced back to a 1927 typhoid epidemic in Montréal caused by contaminated milk ((9)). In 1938, Ontario became the first Canadian province to ban all sales of raw milk ((9)). In 1991, Canada’s *Food and Drug Regulations* officially banned the sale of raw milk due to concerns over food-borne illnesses like *E. coli* and severe sequelae from HUS following raw milk consumption ((9)). Health Canada data shows that mandatory pasteurization has been linked with a decrease in the number of food-borne illness outbreaks from milk, with 45 linked outbreaks between 1975 and 1982 compared against 7 linked outbreaks between 1998 and 2021 ((10)).

Health Canada establishes regulations and standards through the Canadian Food Inspection Agency relating to the safety and nutritional quality of milk sold in Canada ((10)). The Canadian Food Inspection Agency verifies that milk sold in Canada meets Health Canada’s requirements. Sampling of dairy products is performed by an Agency inspector as part of the Agency’s monitoring and compliance activities to verify any suspected problems of potential health risk to the public at a federal level. Provincially, the Ontario Ministry of Agriculture, Food and Rural Affairs licenses all dairy plants under the *Milk Act* ((11)). Ontario Ministry of Agriculture, Food and Rural Affairs requires all raw milk to be graded before being transported from the farm and prior to being received at a processing plant as part of a thorough quality-control process. To maintain Canadian standards, dairy farmers must be licensed and their farms inspected before receiving authorization to ship milk. They must also follow provincial regulations on food safety, animal care and the environment. In Canada, the federal government regulates pasteurization of milk used in the production of cheese, butter, yogurt and other products ((11)). Health officials state that pasteurization of milk retains all the nutrients and health benefits of raw milk, significantly reduces potential human pathogens and increases milk’s shelf life ((6)).

### Proposed recommendations for practice

In the case presented here, raw milk consumption led to a severe paediatric presentation of HUS. Here are proposed solutions that need to be assessed for efficacy.

### Hygiene and rehydration

Practising good hygiene, such as washing hands before handling food and using clean utensils, is crucial to prevent the spread of contaminants. In cases of suspected raw milk consumption-related illnesses, maintaining adequate rehydration is key for better treatment outcomes.

### Physician awareness

Physicians play a vital role in identifying patients at risk of raw milk consumption. By asking directed questions, particularly about raw milk purchases from farm gate sales or “herd shares,” they can counsel patients about the risks associated with the consumption of raw milk (and milk products) and the symptoms of related illnesses. Further recommendations should highlight the importance of consuming only pasteurized milk products, especially for the immunocompromised, pregnant people and those at extremes of age. Physicians should also recommend the purchase of milk products from grocery stores and checking labels on milk products to ensure it has been pasteurized at a licensed dairy plant.

### Public education initiatives

Targeted messaging: Tailor public education campaigns to address specific demographics and communities where raw milk consumption is more prevalent. Develop culturally sensitive and linguistically appropriate content to effectively reach diverse populations.

Health risks awareness: Clearly communicate the potential dangers of consuming raw milk, such as bacterial infections and food-borne illnesses, especially among vulnerable groups like pregnant people, children and individuals with compromised immune systems.

Benefits of pasteurization: Highlight the numerous benefits of pasteurization in preventing food-borne illnesses and protecting public health. Emphasize that opting for pasteurized milk is a responsible choice for personal and community well-being.

Promote safe handling: Educate consumers on safe milk handling practices, including proper storage, refrigeration and expiration date checks. Encourage adherence to guidelines to minimize risks associated with milk consumption.

Collaboration with health professionals: Engage health care providers in disseminating information about raw milk safety to patients, reinforcing key messages during routine medical appointments.

### On-farm food safety training

Introducing on-farm food safety training programs for raw milk producers can be an effective risk management option. Evaluating the efficacy of these programs can contribute to reducing outbreak rates and enhancing public health. Similar programs have been adopted in the United States and may be a factor in the recent decline in outbreak rates ((5)).

### Research and standards development

Conduct comprehensive research: Undertake in-depth research to understand the prevalence of raw milk consumption, associated health risks, and factors influencing consumer decisions in Canada. These data will serve as the foundation for informed policy-making and public awareness campaigns.

Standards development: Collaborate with regulatory bodies, dairy industry stakeholders and health experts to establish and update stringent standards for milk safety. Regularly review and improve safety guidelines to align with the latest scientific findings and ensure consumer protection.

### Improved surveillance

Current surveillance of raw milk consumption is limited by the clandestine nature of raw milk distribution and the limited reporting of enteric illness associated with consumption. Continued surveillance efforts and outbreak management is critical to identifying specific raw milk and milk product vehicles that can cause enteric outbreaks of *E. coli* and other organisms. For example, larger-scale future food consumption and behavioural risk factor surveys could include questions on raw milk product consumption ((3)).

### Public awareness

Public education campaigns should highlight the health risks of raw milk consumption and promote safe milk handling practices. Raising awareness about the benefits of pasteurization and the importance of purchasing from licensed dairy plants can lead to informed consumer choices.

### Collaborative efforts

Addressing raw milk-related risks requires collaboration among health care professionals, regulatory bodies, dairy industry stakeholders, and the public. By working together, we can protect our communities from the dangers associated with raw milk consumption and promote safer milk consumption practises.

## Conclusion

The popular movement towards raw milk consumption is an ongoing trend that carries preventable risks. Consumption of pasteurized milk and milk products has long been the mainstay in preventing unnecessary food-borne illness and lethal sequelae ((12)). For both physicians and public health agencies, greater surveillance, stricter regulation and enforcement and intensive public education and counselling will create the broad context required to limit raw milk consumption and prevent illnesses in Canada.
